# One generic synonym and one new species of Phlaeothripidae from India (Thysanoptera)

**DOI:** 10.3897/zookeys.786.28332

**Published:** 2018-09-25

**Authors:** Kaomud Tyagi, Devkant Singha, Goutam Kumar Saha, Vikas Kumar

**Affiliations:** 1 Centre for DNA Taxonomy (CDT), Molecular Systematics Division, Zoological Survey of India, Kolkata, West Bengal, India; 2 Department of Zoology, University of Calcutta, West Bengal, India

**Keywords:** *
Dyothrips
*, *
Haplothrips
*, India, new species, synonym.

## Abstract

*Haplothripsshivendraii* Tyagi & Kumar, **sp. n.** is described from Rajasthan state of India. The monobasic Austro-oriental genus *Dyothrips* Kudô is formally synonymised with *Haplothrips*.

## Introduction

The genera *Haplothrips*, *Dyothrips*, and *Plicothrips* belong to tribe Haplothripini in the subfamily Phlaeothripinae, family Phlaeothripidae (Mound and Minaei 2007, Minaei and Mound 2008). *Haplothrips* was erected by Amyot and Serville (1843) for the single species, *Phloeothripsalbipennis* Burmeister, 1836. It is the second largest genus in the family Phlaeothripidae and comprises the two subgenera *Haplothrips* and *Trybomiella*. These are distinguished by the presence or absence of fore wing duplicated cilia, present in *Haplothrips* and absent in *Trybomiella*. The genus currently includes 242 extant species, of which 219 are in *Haplothrips* and 23 in *Trybomiella* (ThripsWiki 2018). From India, 22 Haplothrips species are recorded, 16 in the subgenus Haplothrips and six in *Trybomiella* (Tyagi and Kumar 2016).

*Dyothrips* was first described as a subgenus by Kudô (1974) to include the single species Haplothrips (Trybomiella) cingulatus Pelikan, 1963 from China, and he simultaneously recorded this species from Taiwan (Kudô 1974). However, two further species described from Australia, *Zygothripspallescens* Hood, 1919 and *Watsoniellahelena* Girault, 1928 were later synonymized with *cingulatus* (Pitkin 1973). Bhatti (1995) elevated the status of *Dyothrips* from subgenus to genus to include the single species *Dyothripspallescens* (Hood, 1919). This was based on two morphological characters: incomplete notopleural sutures, and complete mesopresternum. The genus *Plicothrips* Bhatti, 1979 included two species, *Hindsianaapicalis* Bagnall, 1915 from India and *Hindsianacameroni* Priesner, 1934 from Sudan.

The genus *Dyothrips* is closely related to *Plicothrips* by the presence of one sense cone on antennal segment III and incomplete notopleural sutures. However, it can be separated by the presence of two pairs of wing retaining setae in *Dyothrips* and one pair in *Plicothrips*. Furthermore, according to the key to Australian genera of the *Haplothrips* lineage group (Mound and Minaei 2007) *Dyothrips* is distinguished from *Haplothrips* based solely on incomplete notopleural sutures. Those authors pointed out that *Dyothrips* and *Haplothrips* do not differ in the mesopresternum because this structure is completely sclerotised in the type species of *Haplothrips*. Recently, we collected a Haplothripini species from Rajasthan state of India and found the notopleural sutures were incomplete in eight specimens, but complete in four specimens, and in a further specimen this suture was incomplete on the left side but complete on the right side. These ten specimens were all collected from the same locality on the same plant, and this variation suggests that the complete or incomplete condition of these sutures is not robust enough to separate the genus *Dyothrips* from *Haplothrips*. As a result, the genus Dyothrips is formally synonymized under the subgenus Trybomiella of the genus *Haplothrips*.

The objective of the present study is to describe a new species of *Haplothrips* from Rajasthan state of India and to synonymise the genus Dyothrips under the subgenus Trybomiella of genus *Haplothrips*.

## Materials and methods

Holotype and paratypes are deposited in the National Zoological Collections (**NZC**) at Zoological Survey of India, Kolkata. The specimens were collected by beating vegetation over a white tray, and picked by using a camel-hair brush wet in 70% alcohol and stored in -20 °C. The specimens were then mounted onto the glass slides in Canada balsam for identification. Morphological terminology for adult structures mainly follows Mound and Minaei (2007). Photographs and illustrations were taken with a Leica Trinocular Microscope (Leica DM-1000) using Leica software application suite (LAS EZ 2.1.0). The identification was done using available keys (Pitkin 1976; Ananthakrishnan and Sen 1980; Mound and Minaei 2007; Minaei and Mound 2008).

## Taxonomy

### Haplothrips

Taxon classificationAnimaliaThysanopteraPhlaeothripidae

Amyot & Serville, 1843

Haplothrips Amyot & Serville, 1843: 640.
Dyothrips Kudô, 1974: 114. Syn. n.

#### Remarks.

The Austro-oriental genus *Dyothrips* is known by the single species *D.pallescens* Hood, 1919 from China, Taiwan, Thailand, Japan, Australia, Fiji, and India. Because of variation in the notopleural sutures in the new species described below, *Dyothrips* can no longer be distinguished from *Haplothrips*, and they are here formally synonymised. The new combination, *Haplothripspallescens* (Hood, 1919), is established here.

### Haplothrips shivendraii

Taxon classificationAnimaliaThysanopteraPhlaeothripidae

Tyagi & Kumar sp. n.

http://zoobank.org/31D6F646-D7E9-4D29-960B-05A86D23B121

Figures 1 , 2 , 3 , 4 , 5 , 6 , 7 , 8 , 9

#### Diagnosis.

Both sexes macropterous. Body dark brown, fore wing transparent. Antennae 8-segmented, III with two and IV with four sense cones. Head longer than broad, maxillary stylets widely separated, maxillary bridge complete; with one pair of postocular setae, capitate. Pronotal epimeral setae (ep) well developed, capitate, notopleural sutures incomplete or incomplete. Mesopresternum divided into two lateral triangles. Fore wing without duplicated cilia. Fore tarsal tooth small in female and developed in male. Pelta triangular.

#### Description.

**Figures 1.**
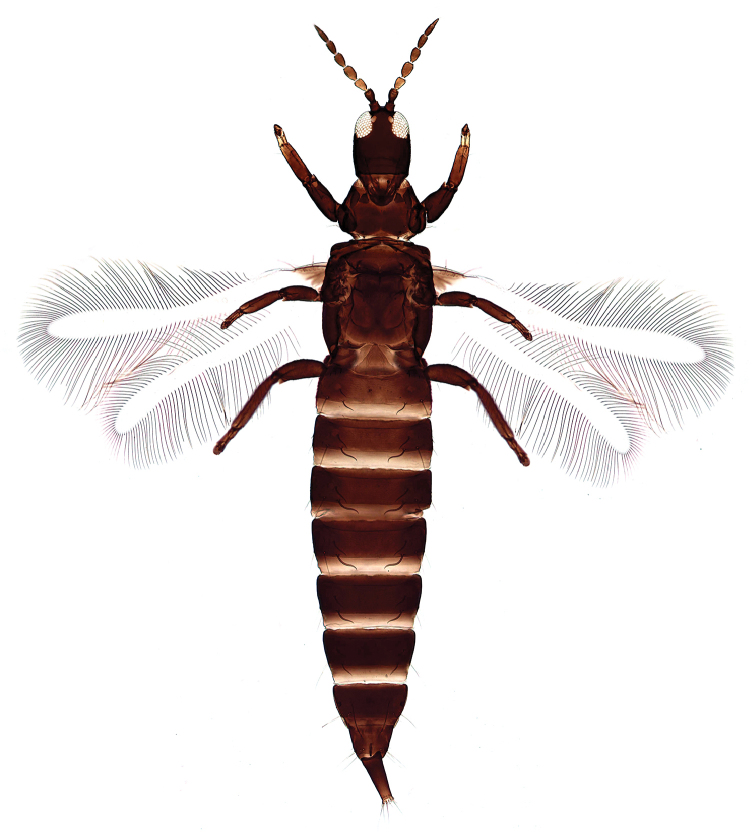
*Haplothripsshivendraii* sp. n.: Female

Female macroptera. Body dark brown, all femora, mid and hind tibiae, mid and hind tarsi brown; fore tibiae light brown, fore tarsi yellow with unguitractor dark, fore wing transparent, shaded with brown basally (Figure 1). Antennae brown except light brown segment III. Head longer than broad, dorsal surface with few transverse striae (Figure 3). Maxillary stylets retracted to postocular setae and one third of width apart, maxillary bridge present; one pair of postocular setae well developed, capitate; eyes enlarged dorsally than ventrally; ocelli present. Antennae 8-segmented; segment II with campaniform sensilla situated apically; segment III with two sense cones, IV with four sense cones, V with three-one-one sense cones situated outer and inner margin of apex, and one small sense cone on apex, VI with two, VII with one sense cone; segment VIII not constricted at base (Figure 4). Mouth cone rounded. Pronotum rectangular, 1.7 times as broad as long, and 0.6 times as long as head; dorsal surface with few striae laterally and posteriorly and many small setae; anteroangular setae (aa) small and blunt apically, anteromarginal (am) and midlateral (ml) setae small, pointed or reduced, posteroangular setae (pa) capitate, little longer than aa and shorter than ep, epimeral setae (ep) well developed, capitate, and longer than posteroangular (pa); notopleural sutures incomplete or complete. Mesonotum dorsal surface with faint transversely reticulate sculpture, median and submedian setae little far from posterior margin; lateral setae expanded at apex. Metanotum weakly sculptured with reticulation, with well-developed median pointed setae. Mesopresternum divided into two lateral triangles (Figure 5). Fore wing with median constriction, without duplicated cilia; sub-basal wing setae arranged in one row, well developed and capitate, and S3 the longest (Figure 6). Fore tarsal tooth small (Figure 3). Pelta triangular in shape, surface with reticulation (Figure 7). Tergites III–VII with 2 pairs of wing retaining setae (Figure 8); tergite IX setae S1 bluntly pointed, S2 and S3 finely acute (Figure 9). Sternites II–VIII with accessory setae. Tube shorter than head, anal setae shorter than tube.

**Figures 2.**
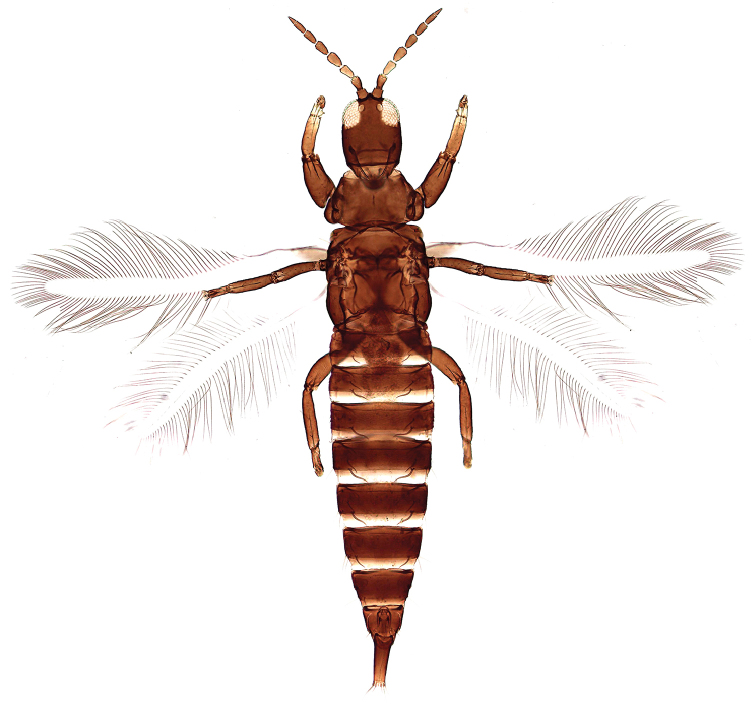
*Haplothripsshivendraii* sp. n.: Male

**Figures 3.**
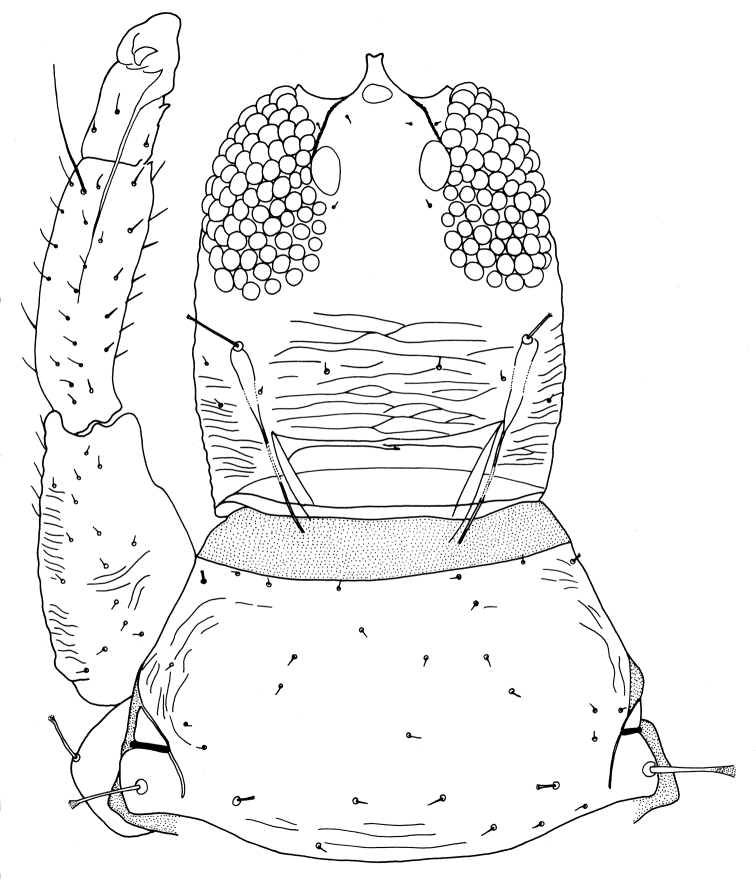
*Haplothripsshivendraii* sp. n.: Head and Prothorax with fore leg

**Figures 4.**
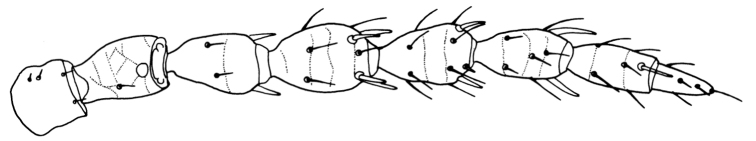
*Haplothripsshivendraii* sp. n.: Antenna, female

**Figures 5.**
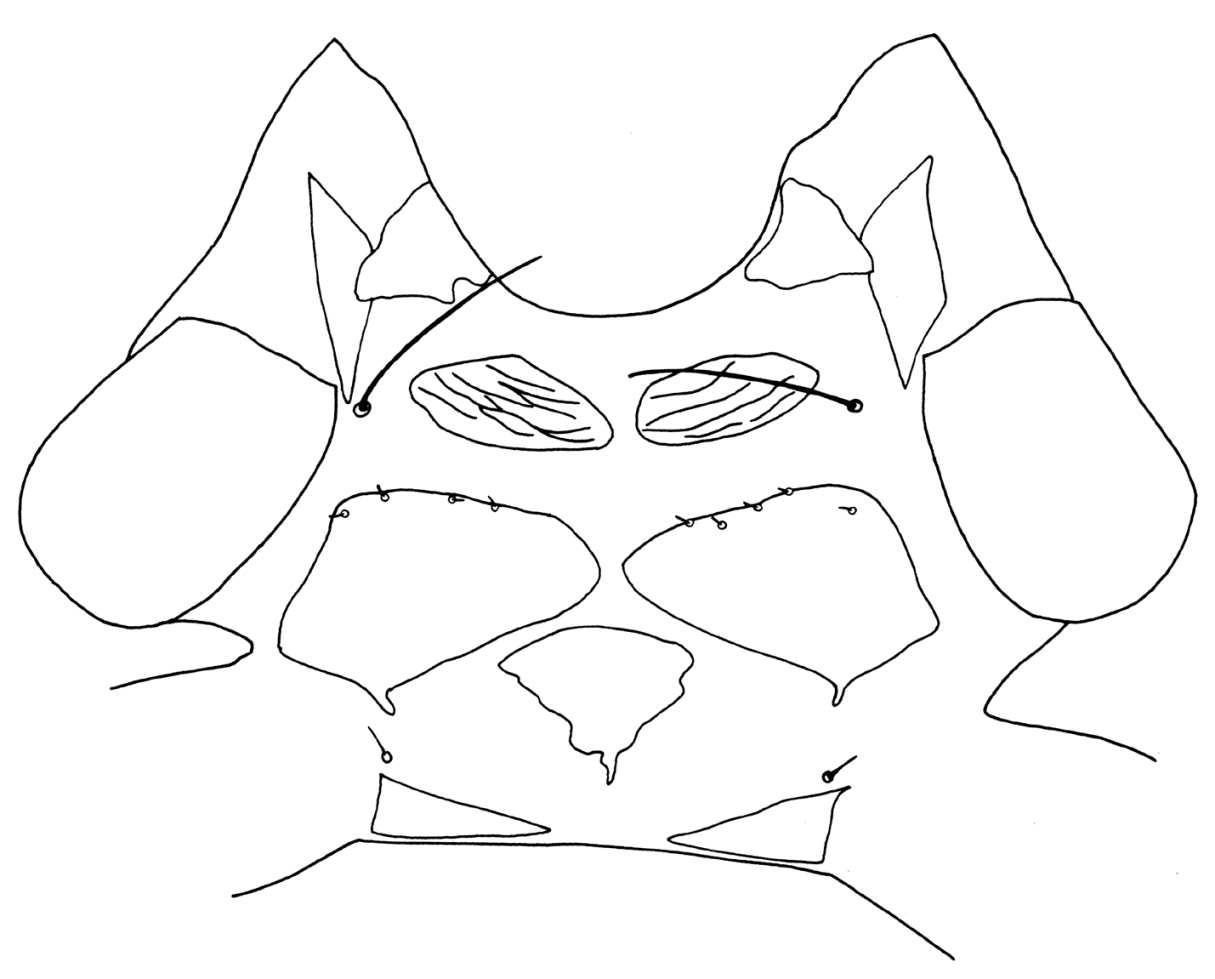
*Haplothripsshivendraii* sp. n.: Prothroax, ventral view, female

**Figures 6.**
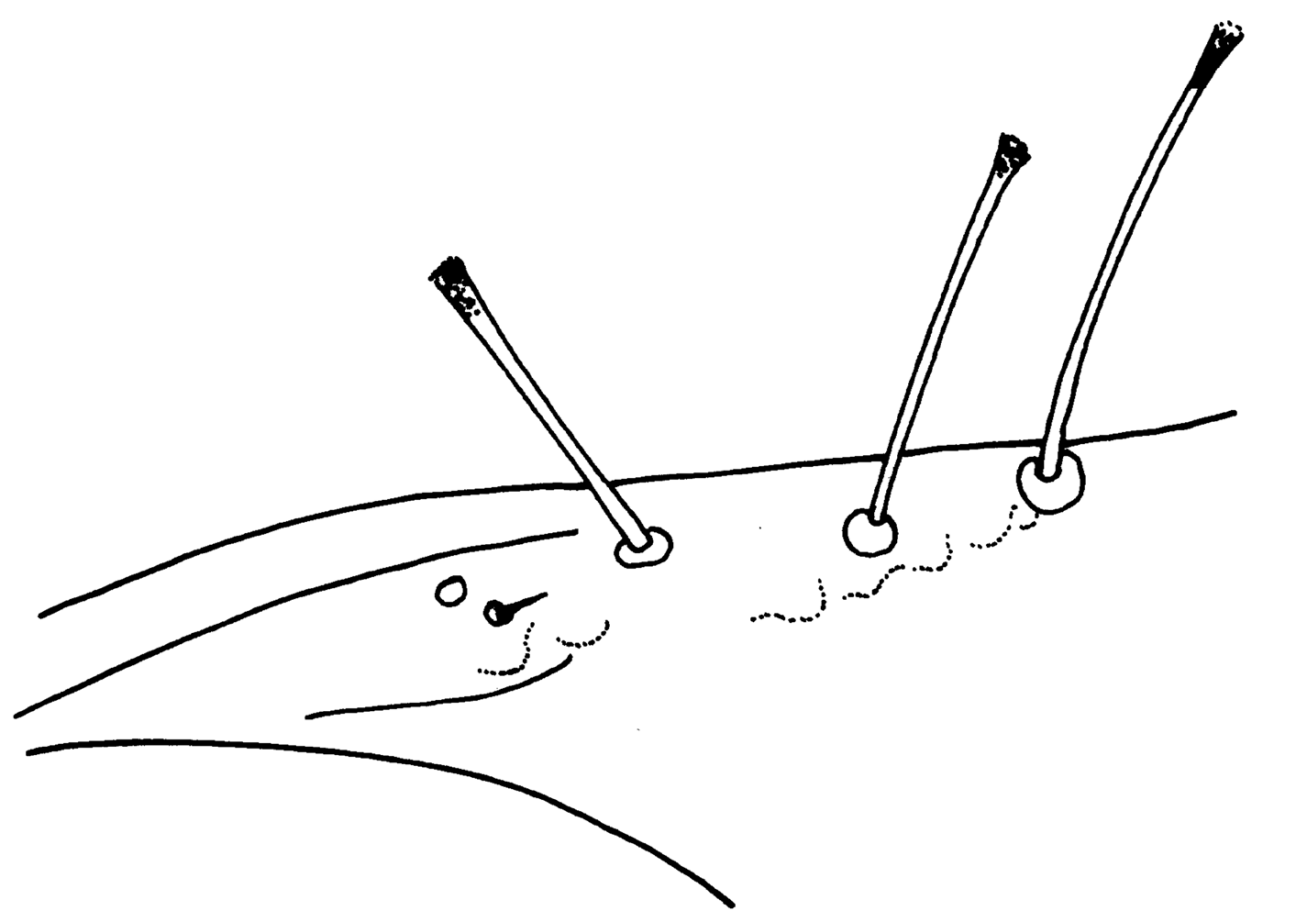
*Haplothripsshivendraii* sp. n.: part of fore wing, female

**Figures 7.**
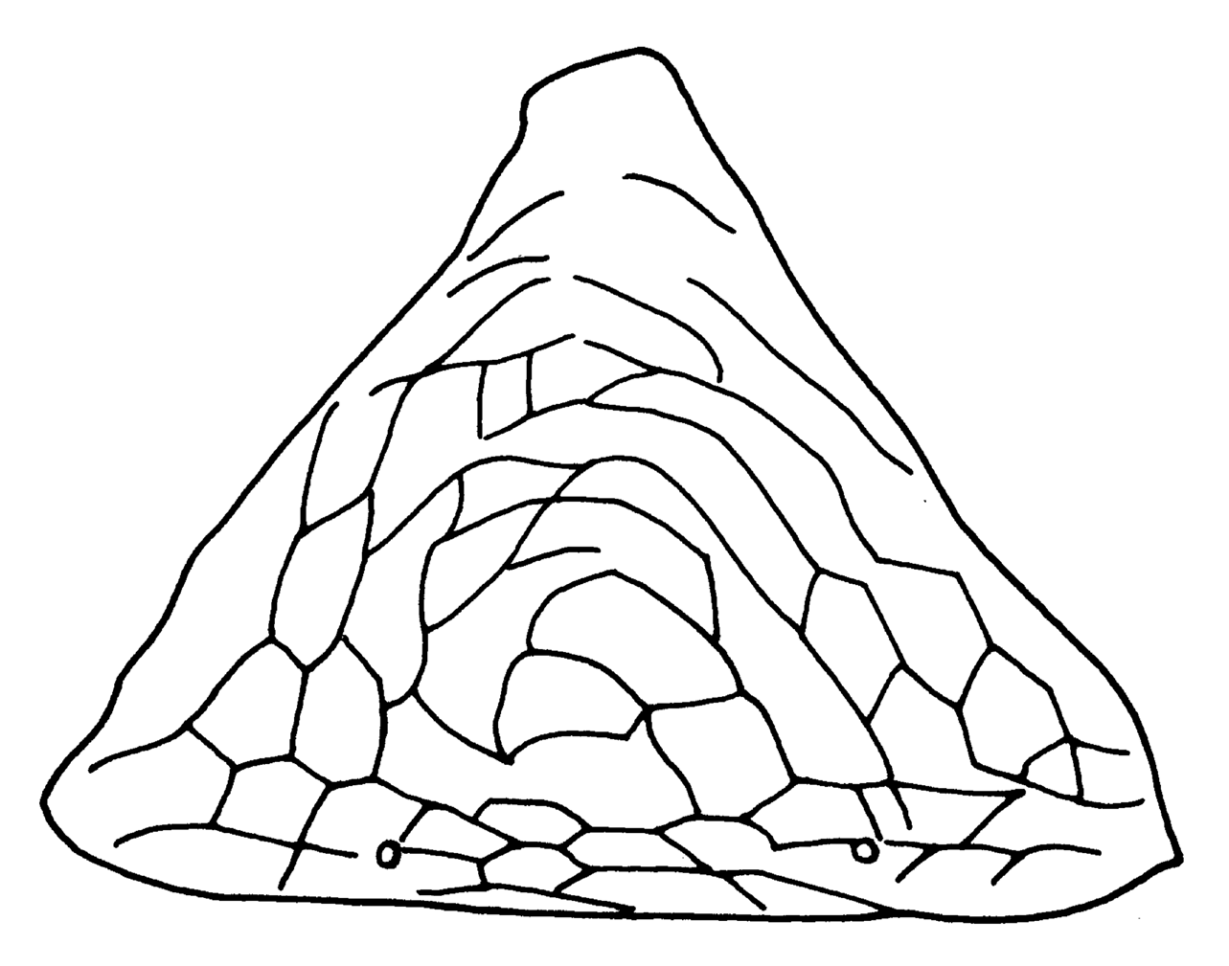
*Haplothripsshivendraii* sp. n.: Pelta, female

**Figures 8.**
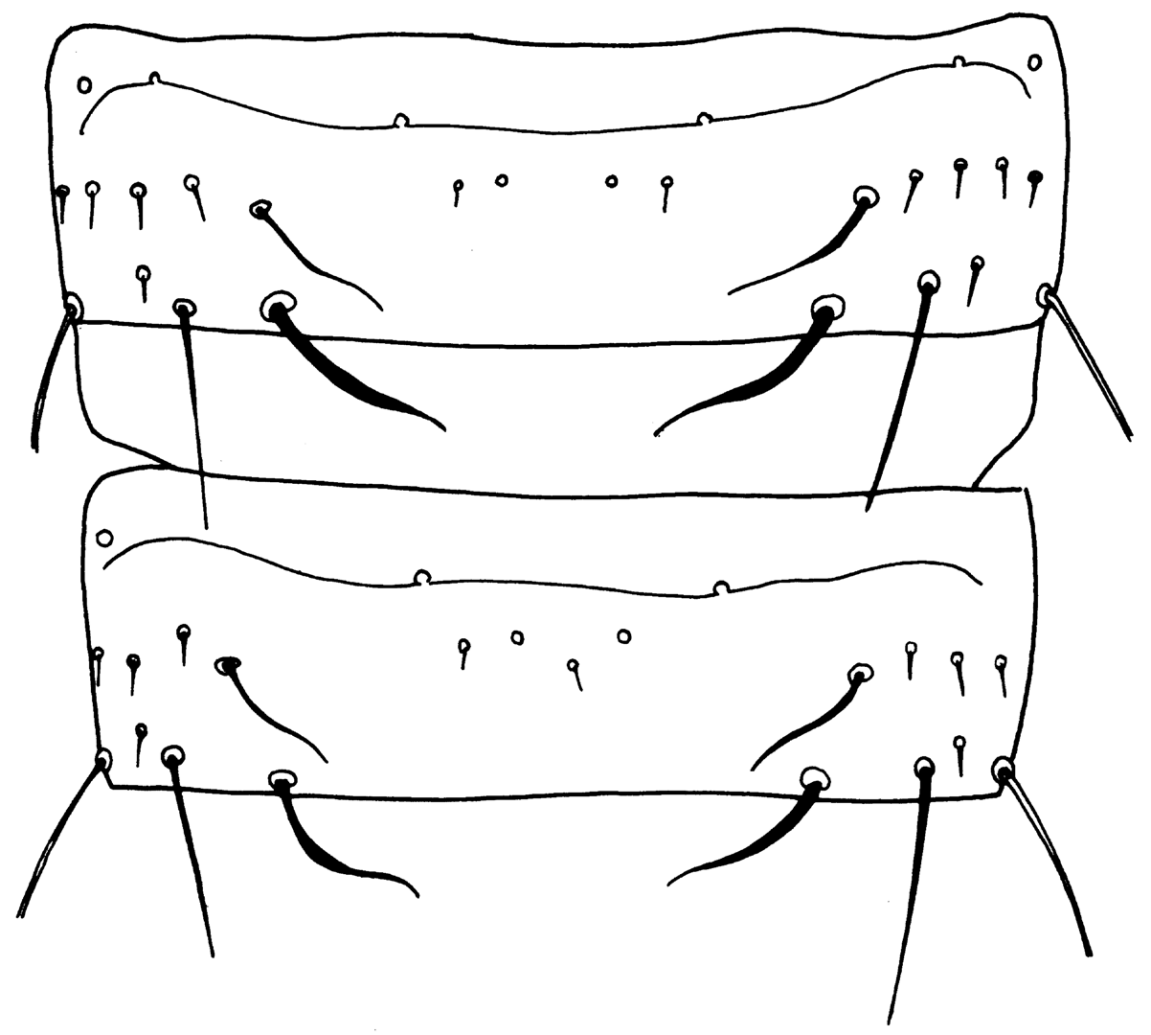
*Haplothripsshivendraii* sp. n.: Tergites IV–V, female

**Figures 9.**
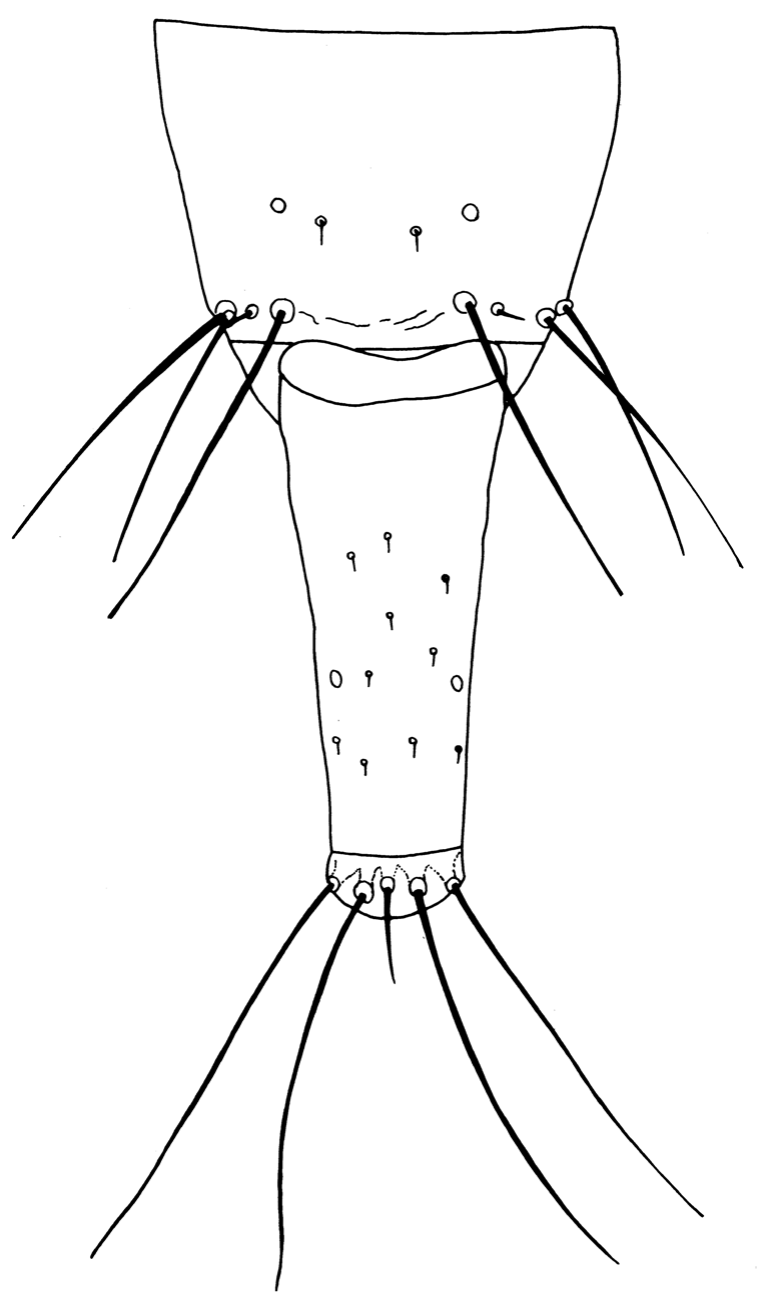
*Haplothripsshivendraii* sp. n.: Tergites IX–X, female

#### Measurements.

(holotype female in microns). Body length 2020; head length 225, width across eyes 170, across cheeks 176, across cheeks just before basal collar 159; eye length 98–100, width 50–55; postocular setae lengths 25–28; pronotum median length 133, width 238, lengths of major setae: pa 10, epim 36–42; pelta length 96, width at base 120; antenna length 318, L(W) of antennal segments I 27–29(29), II 39–42 (29), III 43(28), IV 46–49(32), V 41–43(28), VI 40(23), VII 37(20), VIII 27–29(11); fore wing basal setae length S1 39–40, S2 52–54, S3 62–63; tergite IX length 70; setae S1 102–104, setae S2 87–88; tube length 153, width at base 34, at apex 62; anal setae length 99–117.

#### Male.

Macropterous. Colour and structure similar to female (Figure 2). Fore tarsus with distinct and well developed tooth. Male sternite VII without pore areas.

#### Material studied.

Holotype female, **INDIA**: Rajasthan, Jodhpur, Desert Regional Centre, ZSI, collected from grass, 1.i.2015, (Reg. No.9542/H17), Coll. Shivendra Kumar Singh, in National Zoological Collections (NZC). Paratypes: 8 females 4 males, taken with holotype (Reg. No. 9543/H17 to 9554/H17).

#### Etymology.

This species is credited to Shivendra Kumar Singh for his keen interest and untiring effort for thrips collection dating back to his childhood.

#### Distribution.

India (Rajasthan).

#### Remarks.

This new species is similar to *Haplothripspallescens* in having incomplete notopleural sutures in ten specimens. It can be distinguished by the body colour, which is brown in the new species but bicoloured in *pallescens*; the pronotal anteroangular and anteromarginal setae are not developed in *shivendraii*, but well developed and capitate in *pallescens*. There are two sense cones on antennal segment III and four on IV in *shivendraii* but one sense cone on III and three on IV in *pallescens*.

According to Indian key to the order Thysanoptera (Ananthakrishnan and Sen 1980), the new species is similar to *Haplothripsnigricornis* (Bagnall) by the length of the pronotal midlateral setae and anteroangular setae. It can be distinguished by the yellow fore tarsus (yellowish brown in *nigricornis*), light brown fore tibia (fore tibia brown with slightly paler apex), presence of four sense cones on segment IV (4+1 in *nigricornis*); pronotum with posteroangular setae developed (reduced in *nigricornis*); and maxillary stylets are more widely separated in *shivendraii* than *nigricornis*.

## Supplementary Material

XML Treatment for Haplothrips

XML Treatment for Haplothrips shivendraii
